# Special Issue: Microbiota–Gut–Brain Axis

**DOI:** 10.3390/microorganisms10020309

**Published:** 2022-01-28

**Authors:** Mark Obrenovich, V. Prakash Reddy

**Affiliations:** 1 Veteran’s Affairs Medical Center, Case Western Reserve University, Cleveland, OH 44106, USA; 2 Department of Chemistry, Missouri University of Science and Technology, Rolla, MO 65401, USA

There is emerging evidence that human health and disease are modulated by the microbiota and their various metabolites, formed through intestinal and gut bacterial metabolism [1,2,3,4,5]. Disruption of the gut–brain axis adversely affects human health, resulting in a variety of pathologies, and in particular, the gut–brain axis disruption is also involved in various neurological disorders, including Alzheimer disease (AD), Traumatic Brain Injury (TBI), multiple sclerosis (MS), and Parkinson disease (PD). The gut microbiota and their metabolites play a significant role in the regulation of cognition and thus therapeutic strategies based on microbiota-derived metabolites may have a profound impact on the future of medicine, as related to central nervous system (CNS) related disorders. Moreover, these metabolites are also important in a variety of other diseases, ranging from inflammation, cardiovascular disease, and obesity to diabetes [6,7]. The gut and intestinal bacterial metabolites of the dietary polyphenolic compounds and other plant-based lignins play an important role in the modulation of human health through gut–brain axis. The dysbiosis of the microbiota and these deleterious or diminished bacterial metabolites have implications in the onset of various neurological disorders and other diseases [8,9].

This Special Issue addresses the current developments in this ever-expanding area and is focused on the diverse roles of microbial and human metabolites have on the microbiota–gut–brain–neuroendocrine axis. The following articles in this special edition highlight the microbiota–gut–brain axis and its effect on human health and disease. Bai, Zhang, and coworkers [10] have assayed the association of the gut microbiota with the temperament for infants, focusing on the abundance of the gut microbiota of the Hungatella and Bifidobacterium genera. Song and coworkers [11] have investigated the chocking and performance behavior in the athletes as a function of the gut microbiota and conclude that the athletes’ performance is positively correlated with the yogurt bacterial species, consisting of the Bifidobacterium and Lactobacillaceae bacterial species. Elkins and coworkers [12] have developed polymerase chain reaction (PCR) high-resolution melt (HRM) assays for detecting and identifying bacterial species, such as Escherichia Coli, Bacillus cereus, and Vibrio parahaemolyticus, which are abundant in the human body and on the skin surfaces. Obrenovich and coworkers [13] have outlined the effects of the microbiota in the prosaic foods on the microbiota–gut–brain axis–heart shunt, as related to its connection with the neurological diseases, including Alzheimer disease (AD). Obrenovich and coworkers [14], in a concept paper, provided the positive correlation of the antioxidant-rich, polyphenol-based diet, consisting of various fruits, and vegetables on the brain and heart health. Vasquez and coworkers [15] have investigated the effects of the gut microbiota on the attention deficit/hyperactive disorder (ADHD), an increasingly common neurodevelopmental disorder. Obrenovich and coworkers [16] in a hypothesis paper discuss the effects of the antibiotics, and the microbiota-derived drugs (“bugs as drugs”) on the microbiota–gut–brain (MGB) axis. Adeli and coworkers [17] have reviewed the role of the gut microbiota in neuroendocrine regulation of nutrient metabolism, via the microbiota–gut–brain–liver axis. Reddy and coworkers [18] have outlined the role of the polyphenolic compounds-derived gut bacterial metabolites in health and disease, focusing on their protective effect on the traumatic brain injury (TBI), Alzheimer disease (AD), and Parkinson disease (PD). Prochazkova and coworkers [19], in a clinical study, have investigated the gut microbiome and microbial metabolites on the anorexia nervosa (AN) pathology, although with minimal effects on the patient clinical outcome. 

It is hoped that this special edition would stimulate further exciting research in the interdisciplinary areas of medicinal chemistry, neuroscience, and drug discovery, and would lead to the mechanistic understanding of the microbiota-gut-brain connection and thereby would lead to the development of efficient therapeutics for various human pathologies.

## Data Availability

Not applicable.
